# Improving general practitioners’ approaches to functional somatic syndromes: a pilot training program with a focus on compassion and communication

**DOI:** 10.1186/s12909-024-06619-0

**Published:** 2025-01-18

**Authors:** Gonthier Ariane, Linder Audrey, Sommer Johanna, Despland Jean-Nicolas, Ambresin Gilles

**Affiliations:** 1 Institute of Psychosomatic and Psychosocial Medicine for Romandie (IMPPRo), Lausanne, Switzerland; 2 School of Health Sciences (HESAV), University of Applied Sciences and Arts Western Switzerland (HES-SO), Lausanne, Switzerland; 3 Department of Psychiatry, University Institute of Psychotherapy, Lausanne University Hospital (CHUV), Lausanne, Switzerland; 4 Institute of Primary Care, Faculty of Medicine, Geneva University, Geneva, Switzerland

**Keywords:** Psychosomatic teaching, Compassion meditation, Functional somatic syndromes, Medically unexplained symptoms, Medical education

## Abstract

**Background:**

Functional somatic syndromes are common in primary care and represent a challenge for general practitioners (GPs), with a risk of deterioration in the doctor-patient relationship, and of compassion fatigue on the part of the physician. Little is known about how to teach better management of these symptoms.

**Methods:**

The aim of our scientific team was to develop a training session about functional somatic syndromes for GPs, with the objective to improve the therapeutic attitude of the participants. The first session of the training was constructed as a pilot session, followed by a qualitative study to complete content validation. The educational framework of the training session is multimodal and includes theory on the pathophysiology of functional somatic syndromes, communication skills, and introspective learning including an introduction to compassion meditation. 20 physicians attended the pilot training session. 10 of them participated in the qualitative study. The qualitative study consisted of five individual semi-structured interviews and one focus group of five persons, investigating the impact of the training session on the clinical practices, as perceived by the participants. The interviews were analysed through an inductive method inspired by Malterud’s systematic text condensation strategy.

**Results:**

We identified three main themes in the responses of the participants: (1) the crucial issue of putting a name to chronic psychosomatic suffering; (2) the importance of self-compassion for physicians; (3) changes in therapeutic attitude fostering a reconciliation between “self” and “care”. Participants expressed a need for more regular meetings of this type. The opportunity to share their negative feelings about therapeutic relationship within a peer group, with compassionate supervision of the trainers, seemed to play an important role in the improvement of their self-compassion

**Conclusion:**

A multimodal teaching session seems to help the physicians to feel more comfortable and competent when treating patients with functional somatic syndromes. Including compassion meditation in the teaching seems a promising tool to prevent compassion fatigue.

**Supplementary Information:**

The online version contains supplementary material available at 10.1186/s12909-024-06619-0.

## Introduction

Functional somatic syndromes are common in primary care [1, 2]. These disorders are also known as “medically unexplained symptoms” or sometimes “psychosomatic disorders” and were formerly classified as “somatoform disorders” or “somatization” in the DSM-IV (now “somatic symptoms disorders” in the DSM-5). Researchers cannot reach a consensus about their terminology [3]. In this article, we refer to “functional somatic syndromes” as defined by Barsky and collaborators [4], i.e. chronic unexplained somatic symptoms, which can be organized into slightly different constellations with different names and various etiological hypotheses, but sharing a common phenomenology in the way they become chronic, disabling and in the type of reaction they elicit from clinicians.

These syndromes present a challenge for physicians, who are confronted with their limitations in managing medical symptoms [5, 6]. In clinical practice, psychosomatic suffering is frequently difficult to classify, patients seemingly presenting with symptoms in many organic and psychologic systems, sometimes changing from one consultation to another. As there is no scientific consensus to explain the pathophysiology of these symptoms, general practitioners (GPs) often have great difficulty answering patients when asked about the origin of their suffering [7], which can be noxious for the doctor-patient relationship [8]. Unlike other chronic diseases, where therapeutic failure can be attributed to the disease considered incurable, therapeutic failure in functional disorders is often experienced as a personal failure on the part of the physician, or ill will on the part of the patient. This can be a source of counter-attitudes among physicians when facing this type of problems [9], which may lead either to the rejection of these patients, sometimes considered as “*heartsink patients”* [10], or to physician burnout [11]. Insecurity of diagnosis when unexplained symptoms do not resolve spontaneously can also drive to costly escalation of specialized tests [9, 12].

Although recent guidelines on good management of functional somatic syndromes emphasize the importance of strengthening the doctor–patient relationship [13] little is known about educational methods that can help clinicians feel more comfortable in the relationship with patients suffering from functional disorders [14–16]. A couple of training programs are focused on communication skills [17, 18], but none to our knowledge is specifically addressing the counter-attitudes of clinicians facing patients with functional somatic syndromes.

Based on the premise that these patients would be better cared for if the physician felt more competent and less exhausted, we brought together a multidisciplinary team to develop a training session on functional somatic syndromes for general practitioners in private practice.

We hypothesized that in addition to good communication skills, clinicians also need strategies to protect against the risk of compassion fatigue, the exhaustion of caregivers overwhelmed by their dedication trying to help chronically helpless patients [11], supported by the findings that affective sharing is a risk factor for compassion fatigue [19, 20]. In the present study, we refer to compassion as understood in a neuroscientific model of empathy developed by Decety [21], where compassion is considered as the motivational dimension of empathy (also called empathic concern, which refers to the motivation to care for someone’s welfare), besides its cognitive dimension (the ability to step back from what others are experiencing, also called perspective taking) and its emotional dimension (or affective sharing). Previous studies suggest that compassion could be a protective factor against physician burn-out [19, 22]. Many studies suggest that compassion can be trained, with a positive effect on emotion regulation [23–25]. A recent systematic review of educational interventions showed that different educational concepts can be useful for teaching compassion to undergraduate medical students (communication, mindfulness, early clinical exposure, technology-assisted learning, comics, arts and culture), but was unable to identify the superiority of one strategy over another [26].

Therefore, we created a multi-modal educational format, combining theoretical, while interactive, lectures on functional syndromes with experiential learning, including role-playing and discussion of real clinical cases. A specific part of the training was devoted to the issue of empathy and the risk of compassion fatigue, including an introduction to compassion meditation. The impact of the training program, as perceived by the participants, was then evaluated through a qualitative study.

## Methods

### Process and steps in the development of the program

The program development process was conducted in accordance with the ADDIE model [27], which entails five sequential steps: analysis, design, development, implementation, and evaluation. A series of steps was undertaken to define the objectives and scope, to develop a content outline, and to create an evaluation framework. Three members of the research team (AG and JS, experienced GP clinicians and trainers in psychosomatic disorders, JND experienced psychiatrist and trainer in psychosomatic disorders) are experts in teaching psychosomatic disorders. Based on their previous experience with general physicians as learners and on existing literature [28], they identified the learning objectives, established the scope of the course (knowledge skills and attitudes for accompanying psychosomatic disorders), and developed the content outline. The three experts evaluated the content relevance, coverage and validity reaching a consensus. The training was then delivered to a small group of GPs as a pilot program. After the pilot training, feedback was collected by questionnaires and semi-structured interviews (5 individual interviews and one focus group) to complete the content validation.

### Training program

We developed a training program divided in three sessions of two days each, building most of the teaching on theoretical interactive discussions and role plays grounded on real clinical cases brought in by participants. Each participant was asked to provide a clinical story in one of the following 6 categories: (1) chronic pain; (2) chronic fatigue syndrome or fibromyalgia; (3) functional digestive disorders; (4) functional neurological disorders; (5) health anxiety, and (6) chronic depression. Chronic depression is included in the teaching because it has an important correlation with chronic physical symptoms [29, 30]: the impact of chronic physical symptoms is worse in the presence of depression; and a depressed mood is frequently found in patients presenting with chronic unexplained symptoms. Moreover, in GPs’ representations, there is a great overlap between patients suffering from chronic depression and patients in complex psychosocial situations complaining of multiple somatic symptoms [29].

The first session is devoted to the specific question of diagnosis, as this is often the first problem clinicians face when trying to treat a functional somatic syndrome using their usual clinical thinking, which implies the need to determine a diagnosis for the symptoms before choosing a therapeutic strategy. Psychosomatic suffering is by definition very difficult to classify in diagnostic categories. This first session is comprised of theoretical overviews of the most recent scientific understanding of the physio/psychopathology of functional somatic syndromes, with the goal of restoring participants’ sense of medical competence. After the presentation of the theory, role-plays based on the clinical cases are enacted to practice communication skills, with the objective to co-construct a diagnosis with the patient.

The second session is devoted to the issue of empathy and acceptance of psychosomatic suffering, as an empathetic therapeutic relationship has been widely described as a key component of good management of functional somatic syndromes, but at the same time as a risk of compassion fatigue, particularly when symptoms are chronic. Based on recent literature [18–24], we decided to explore compassion meditation as a tool to teach empathy and prevent compassion fatigue. First, theoretical insights are given on the different aspects of empathy and the risk of compassion fatigue. Then participants take part in a one-day initiation to compassion meditation. And another type of role-playing (psychodrama role-playing) is also used to help physicians deepen their understanding of the inner perceptions and emotions of patients living with chronic functional somatic syndromes.

The third session is designed to give participants the opportunity to practice their communication skills in co-constructing a care plan with patients, once the issues of diagnosis and empathy have been addressed. The co-construction of the care plan is also acted out in role-playing in pairs, with the aim to improve the communication skills of the physicians, but also to allow them to experience the emotional response and compassion elicited by the confrontation to the patient ailments through the identification either with the therapist or with the patient.

The program was developed with the objective of fostering a culture of learning in an environment that is safe and non-judgmental. Teaching team is comprised of 10 senior clinicians with special interest in the care of patients with chronic psychosomatic disorders (Table 1).

**Table 1:** Teaching team

	Gender	Age	Specialized in	Years since graduation
1	M	59	Gastro-enterology	27
2	M	64	Psychiatry	39
3	F	45	Neurology	21
4	F	47	General internal medicine	22
5	F	55	General internal medicine	29
6	M	62	Psychiatry	37
7	M	51	Psychiatry	20
8	F	43	Psychiatry	18
9	F	65	General internal medicine	40
10	F	49	Insurance medicine	24

Learning objectives and educational framework are summarized in Table 2.

**Table 2:** Learning objectives and educationnal framework

Learning Objective			Title	Thema	Content
**Diagnosing functional somatic syndromes**	DAY 1	AM	Introduction	Diagnosing functionnal disorders	• Collection of experience and expectations of the participants • Theoretical overview
			Elaboration of a case study	Functional digestive disorder	• Case presentation by a participant • Theory about physiopathology and psychopathology by the specialised teacher • Interactive discussion with the group about difficulties in assessing the diagnosis
		PM	Elaboration of a case study	Chronic fatigue	
			Elaboration of a case study	Functionnal neurologic disorder	
	DAY 2	AM	Elaboration of a case study	Health anxiety	
			Elaboration of a case study	Chronic pain	
		PM	Elaboration of a case study	Chronic depression	
			Workshop	Disability report for social insurance	Interactive discussion about how to complete the disability report for one case study
**Welcoming chronic psychosomatic suffering with empathy**	DAY 3	AM	Introduction	Empathy and compassion fatigue	Theoretical overview Experiential workshop of empathic reflecting
			Psychodrama role play	Welcoming emotions	2 groups of 10 people. In each group one physician participant plays the role of his/her patient in front of the group. Another participant plays the doctor. The focus is on what the doctor/patient are experiencing : patient, doctor and the rest of the group is asked about the emotions, the sensations and/or the metaphors they could perceive in the role-play
		PM	Experiential workshop	Initiation to compassion meditation	• Preparatory techniques: physical centering • Theoretical support of compassion • Practical body experience of compassion • Introduction to self-compassion • Introduction to compassionate meditation
	DAY 4	AM	Experiential workshop	Initiation to compassion meditation	• Preparatory practice: mindfulness meditation • Practical body experience of compassion • Practice of self-compassion • Deepening the practice of compassionate meditation
		PM	Psychodrama role play	Welcoming emotions	See DAY 3
			Closure	Links between physician’s perceptions and diagnostic categories	Interactive discussion
Co-constructing a care plan* with the patient	DAY 5	AM	Introduction	Treating functionnal disorders	• Collection of experience and expectations of the participants • Theoretical overview
			Elaboration of a case study	Functional digestive disorder	• Case presentation by a participant • Theory about treatment options by the specialised teacher • role-playing in pairs: treatment implementation • Interactive discussion with the group
		PM	Elaboration of a case study	Chronic fatigue	
			Elaboration of a case study	Functionnal neurologic disorder	
	DAY 6	AM	Elaboration of a case study	Health anxiety	
			Elaboration of a case study	Chronic pain	
		PM	Elaboration of a case study	Chronic depression	
			Workshop	Disability report for social insurance	Interactive discussion about how to complete the disability report for one case study
			Final closure		Interactive discussion

* The words “care plan” were chosen instead of “treatment plan” to emphasize the idea that the aim of medical follow-up is to take care of the patients in the long term and help them live a better life despite their medical condition, rather than hoping for short-term resolution of symptoms (“care rather than cure”)

### Research methods

A qualitative study was designed to evaluate the impact of this program on clinical practices, as perceived by the participants. A qualitative approach allows for an in-depth understanding of the impact of the training program as it captures subjective impressions and real-life applications. This approach is based on the assumption that subjective experiences in training are key and meaningful to change, as they anchor new attitudes and allow for a more open discussion with patients with chronic functional disorders. The study protocol was built taking into account the COREQ checklist [31]. The research team included two psychiatrists (JND and GA), two general practitioners (JS and AG) and a sociologist specialized in qualitative research in mental health (AL). Apart from the sociologist, all members of the research team were also involved in creating and/or teaching the training program.

**Feedback collection and outcomes from the training program.**

The research team developed a semi-structured interview guide aiming to explore the two main focus of this study, namely: participants’ motivations to attend this course and the changes they could perceive after the session in their relationship with patients suffering from chronic psychosomatic conditions, both for the individual interviews (See Supplementary material 1) and for the focus group (See Supplementary material 2). Regarding the individual interviews, the main topics covered were: (1) Professional background and interest in chronic psychosomatic suffering; (2) Discovery, interest and experience of the course; (3) Current management of chronic psychosomatic suffering, and; (4) A summary question. As for the focus group, the key questions included: (1) What were your expectations when you signed up for this course? What made you want to enrol?; (2) Looking back over the three training weekends, how did you experience them?; (3) As participants reported a list of important concepts from the training in their evaluation forms, they were also asked:. To what extent are these concepts still important to you, two year after the course? Have these concepts led to changes in: your ways of diagnosing chronic suffering?; your relationships with your chronically suffering patients? your clinical practice? (4) In the training evaluation, we asked you what changes might occur in your clinical practice as a result of the training. What is the situation today? Have you actually observed these changes?, Did your understanding of the term ‘psychosomatic’ change after this training? Have you observed any other changes? Do you still use compassionate meditation today?, and; (5) Overall, do you now feel more comfortable diagnosing and managing patients with chronic psychosomatic suffering? Helping questions to explore the main parts were also developed and presented in Supplementary material 2.

We estimated that 10 participants would be sufficient to achieve data saturation, which was confirmed by the results. The 20 participants of the first session of the program were contacted by email, and 10 of them agreed to participate in the study. Five individual interviews (mean duration of one hour) and a focus group of 5 persons (duration of 1.5 h) were conducted almost two years after the teaching session. The sociologist (AL) conducted all interviews, audiorecorded and then transcribed them. None of the participants had met AL before. The individual interviews were conducted at the physician’s office, while the focus group was held at the Lausanne University Hospital. The focus group was used to collect a global appreciation of what helped the participants during the training session. It was completed by individual interviews, which allowed to go deeper into participants’ personal experience. As the answers did not differ significantly between the focus group and the individual interviews, they were analyzed together.

**Analysis process.**

We used an inductive method inspired by Malterud’s *systematic text condensation strategy* [32]. To limit potential bias in the interpretation of the data and the conclusions drawn, we employed a triangulation approach, involving multiple evaluators from diverse professional backgrounds: two psychiatrists, two general practitioners, and one sociologist. This diversity in expertise helped ensure a more comprehensive and balanced interpretation of the findings. The first three individual interviews were coded separately by three members of the research team (AG, AL, JS). Research team members read through the transcripts to identify preliminary themes independently and then sorted them into a coding tree constructed by discussion. The discussion continued with further condensation until consensus was reached on primary and secondary themes. The remaining two individual interviews and the focus group interview were coded by AL and AG, using cross-check validation of themes identified in the coding tree.

Throughout the transcript analysis, several consensus meetings were held among the evaluators to incorporate their varied perspectives. These discussions were iterative, continuing until a collective understanding of the data emerged, with the goal of reaching a shared interpretation. Additionally, a continuous comparison approach was employed, where emerging themes were systematically compared to the overall dataset to prevent premature conclusions based on individual evaluators’ subjective impressions. Although not formally documented, reflexivity was strongly encouraged among evaluators during these discussions, prompting them to critically reflect on their own potential biases and preconceptions as they analyzed the transcripts.

## Results

Twenty physicians participated to the first session of the training program. Most of the participants in this pilot training session were primary care practitioners working in urban private practice, and most were already engaged in some form of psychosomatic training (Table 3).

**Table 3:** Participants to the training session

	Age	Gender	Years of practice	Type of practice	Practice site	Previous psychosomatic training
1	40	F	13	General practionner	Urban private practice	yes
2	61	M	30	General practionner	Urban private practice	yes
3	36	F	11	General practionner	Rural private practice	yes
4	43	F	18	General practionner	Urban private practice	yes
5	43	F	15	Endocrinology and diabetology	Hospital	yes
6	44	F	20	General practionner	Urban private practice	yes
7	48	F	23	General practionner	Urban private practice	yes
8	44	M	16	General practionner	Urban private practice	yes
9	38	F	13	General practionner	Urban private practice	yes
10	32	F	7	Training to become general practionner	Medical Clinic	yes
11	39	H	10	General practionner	Medical Clinic	yes
12	45	F	21	Anesthesiology, hypnosis	Urban private practice	yes
13	35	F	7	Training to become general practionner	Medical Clinic	yes
14	38	F	13	General practionner	Medical Clinic	no
15	51	F	24	General practionner	Urban private practice	yes
16	46	F	21	Anesthesiology, hypnosis	Urban private practice	yes
17	32	M	8	Training to become general practionner	Medical Clinic	no
18	42	F	14	General practionner	Rural private practice	yes
19	55	M	28	General practionner	Urban private practice	no
20	63	F	36	General practionner and homeopathy	Rural private practice	yes

All study participants viewed the training session as very positive overall, helping them to better manage chronic psychosomatic patients. Three major themes were identified:

- the crucial issue of putting a name to chronic psychosomatic suffering.
- awareness of the importance of self-compassion for physicians.
- changes in therapeutic attitude fostering a reconciliation between “self” and “care”.

Main components et sub-components of the results are shown in Table 4.

**Table 4:** Main components and sub-components of the results

The crucial issue of putting a name to chronic psychosomatic suffering	understanding physiopathology and psychopathology
	patient reassurance
	moving from an attitude “searching for a cause” to an attitude “searching for solutions to live better with the health problem”
	Better ability to identify a clinical syndrome
	Functional diagnoses are not scientifically solid, only a starting point for psychosomatic discussion with the patient
The importance of self-compassion for physicians	benefits of peer sharing
	understanding the importance of self-care
	the need for ongoing training in psychosomatics (including peer-group discussion and supervision)
Changes in therapeutic posture fostering a reconciliation between “self” and “care”	taking a different consultation pace
	better attunement with the patient
	better ability to define their own limits
	feeling more confident to talk about psychosomatic conditions
	modifying their therapeutic objectives
	increased self-confidence and sense of competence
	feeling more comfortable during consultations, with more pleasure
	shared humanity: doctors are human beings like everyone else

### The issue of putting a name to chronic psychosomatic suffering

During the interviews, the participants said they appreciated the theory about the diagnostic criteria of the different categories and agreed that this effort to “put a name on the illness” was helpful. Most of them reported feeling more comfortable when talking about the symptoms with the patient, and thought that the patient felt better understood in that way. Some of them reported using the pathophysiological hypotheses as a help to destigmatize psychosomatic problems, and to differentiate the person from the disease. Some participants also said that the training program had helped them believe in the reality of the disease, or to think about certain diagnostic categories that they otherwise would not have considered.

Another benefit identified by the participants was the reassuring potential of the functional diagnoses, helping both patient and doctor to feel more secure that no serious disease had been neglected, and thus avoiding unnecessary investigations. As well, participants acknowledged that agreeing with the patient on a medical name for their symptoms made it possible to move from the “search for a cause” phase to a “search for solutions” phase, and thus to mobilize the patient’s resources in the implementation of therapeutic approaches, including psychotherapy.

However, some participants also stressed that these diagnoses are not very solid, and that they are mainly a starting point for a discussion with the patient, bearing in mind that the origin of suffering is probably the same, regardless of the medical name given to it.

### The importance of self-compassion for physicians

When asked about their motivations to undertake this training program, many participants reported feeling helpless, irritated or even exhausted when facing patients with chronic psychosomatic disorders. Some were concerned by their lack of knowledge or skills in this area. Permission to share negative feelings with their peers during the course was experienced as beneficial.

When asked about their practice of compassion meditation, which they had been initiated to during the program, participants reported that they did not practice it anymore. Various reasons were invoked: either they did not like to meditate and preferred to use other means of regeneration, such as gardening or sports; or they had difficulties in self-disciplining for practice; or they did not feel competent enough to practice on their own after a short initiation. However, they were sensitive to the compassionate atmosphere of the program, fostering an awareness of the importance of being compassionate to themselves. Most of them reported to place more importance on resourcing time between their consultations since participating to the training program. Furthermore, they reported having acquired strategies to take care of themselves, such as: identifying the warning signs of compassion fatigue; lowering their expectations of successful treatment; organizing their agenda in order to avoid the close succession of several difficult situations involving chronic psychosomatic disorders.

Participants reported that treating patients with psychosomatic conditions remained a challenge, even after the training program. They also expressed the need of additional sessions of continuous medical training that could give them the opportunity to share their difficulties in the management of psychosomatic patients, emphasizing that this type of training helps physicians to take a step back from their practice, and gives them more energy to continue to take care of psychosomatic patients.

### Change in therapeutic attitude fostering a reconciliation between “self” and “care”

During the interviews, many participants described favorable changes in their therapeutic posture, which helped them feel more comfortable, more competent, and less worried or exhausted when engaging in the care of patients with chronic psychosomatic conditions. Participants also reported they adopted a different consultation pace with a sense of better attunement with the patient and modifications in their therapeutic objectives. Some of the participants claimed that the training did not teach them new therapeutic postures, but rather validated or strengthened favorable attitude they had already adopted with patients suffering from chronic psychosomatic conditions. Favorable therapeutic attitude identified by the participants are listed in Table 5. Overall, changes in therapeutic attitude were experienced as helping to reconcile the need to take care of oneself with the desire to take care of others.

**Table 5:** Therapeutic attitudes fostering a reconciliation between self and care

Changing the pace of the consultation, allowing themselves to be more attentive and available to the patient, with more patience, less fear to lose time, and thus promoting a better listening quality.
Better attunement to the patient, by trying to “get into the patient’s head”, and by taking the time necessary to build trust in the relationship and to co-construct the care plan with the patient.
Feeling more confident to talk about psychosomatic conditions, to make a positive diagnosis of functional disorders, and to suggest connection between bodily symptoms and psychological issues.
Modifying their therapeutic objectives in the direction of “long term care rather than short term cure”.
Increased ability to define their own limits, to say no to expectations of constant availability, and to put an end to a therapeutic relationship when they felt it was turning wrong.

Relevant quotes of the study participants illustrating the 3 major themes are presented in Table 6.

**Table 6:** Quotes

***The crucial issue of putting a name to chronic psychosomatic suffering***	*I can better explain the links between the brain and the intestine*,* and I also use*,* and I think it comes from this training*,* I use this expression that “there is a brain in the belly”. And… this speaks to them*,* yes. Really*,* I have the impression to have the tools to make the diagnosis. To feel confident enough to say: “No*,* stop*,* we’re not doing more”. Because in the end it’s nothing more*,* in fact*,* than labs and clinical exams*,* medical history… And then*,* you don’t necessarily need to to see a gastroenterologist who confirms this*,* if it’s clear. And then*,* afterwards*,* after taking care of these patients*,* after taking care of the symptoms*,* accepting these symptoms*,* we explain that they are more sensitive at the level of their intestine*,* but also that this is not a serious illness that we need to investigate further. That we can try to manage these symptoms by talking. Even if we propose medication that can give some relief. And yeah*,* once this…*,* this diagnosis is understood a little by the patients*,* it helps enormously.*
	*After this I had the opportunity to take care of patients with a diagnosis that we had discussed during this training. In particular*,* a patient with a chronic fatigue syndrome. […]. We looked at the criteria together*,* and we discussed what made sense for him and what did not. And yes then*,* that allowed to actually make the diagnosis. And then it became a real disease*,* in some way. And also it helped to make progress in the management*,* especially by limiting useless additional exams or specialist referrals.*
***The importance of self compassion for physicians***	*And then*,* to stop throwing myself fully into this*,* heart and soul*,* wanting to sort everything out*,* wanting to do everything right. Then*,* to realize that when things don’t go well*,* actually*,* sometimes the problem is not necessarily something I did wrong. But it’s the relationship that is difficult*,* it’s the patient who is sometimes difficult. And then*,* yes*,* it allows me to be a little more indulgent with myself. And to take a little more distance from the emotional load that these patients and these situations may impose.*
	*This compassion meditation*,* I really liked it. I told myself*,* I’m going to do this*,* regularly. This is what I said to myself after the training. Afterwards*,* I must admit*,* I do not really follow it… So*,* I find it hard to put myself*,* on my own*,* in that state of mind*,* to really take the time to meditate on the patient. But sometimes*,* just before a consultation (laughter)*,* I said to myself: Ok*,* I’ll close my eyes (laughter)*,* and have some positive thoughts. And then I think about the patient (laughter). This has actually helped me quite often to face a situation that is difficult. I would say*,* I didn’t really have… I didn’t take the time to do it exactly like we learned. There’s always a lack of time (laughter). But it’s just the approach I think*,* the fact that we… and also the fact that we know we could still do that*,* if it ever goes wrong. That has helped me. And that’s clearly a result of the training.*
	*I think it allowed me to listen to myself. To tell myself that*,* well*,* I had a right to be annoyed*,* by certain patients. I had the right to be tired. Before*,* I think that I wasn’t listening to that. I was looking straight ahead*,* and yes well then*,* you have to*,* you have to*,* you have to….*
***Changes in therapeutic posture fostering a reconciliation between self and care***	*I am already much more at ease*,* I’m enjoying to… to take care of them. In fact*,* it is not any more something that’s imposed on me and then it’s just my job and that’s it. But it really becomes a genuine interest to discover: what can be mobilized or not*,* around these people? And so we are not feeling like we are enduring them. It has completely reversed the position in which which we welcome them*,* in fact. And then*,* as a result*,* the fatigue that we feel. I think*,* effectively*,* it allows me to take… I think the most important thing is the perspective that the patient can have*,* now*,* thanks to the change in my point of view and also the tools that I acquired*,* and then*,* this capacity to make a diagnosis calmly*,* without being completely overwhelmed by the suffering. To tell myself: “well ok*,* I can think about it”. Because that’s the point*,* huh*,* you can’t think any more*,* huh… So*,* there you go. And then to be able to… well*,* it puts the disorder on the table*,* and then there are two of us looking at it. And this is not tiring to do that*,* I mean. So it’s really*,* very important. Lots of enjoyment and lots of interest. It really changed a lot of things in my practice and in my enjoyment to practise medicine.*
	*But yes*,* clearly*,* I feel more comfortable for all the reasons that I’ve already mentioned. But also*,* because I feel better trained*,* today I am much more confident to make a psychosomatic diagnosis*,* much more than two years ago*,* clearly. So*,* the fact that that we were given these tools to dare to name this diagnosis. And also*,* to dare to overcome this fear that we always have of missing something*,* we always have to live with it*,* that’s clear. […] But I think that to have the courage to assert a psychosomatic diagnosis and then being able to move forward with that. Yes I think that’s what this training has given me.*

## Discussion

This paper describes the development and evaluation of a training program designed to improve GPs’ comfort and sense of competence when treating patients with chronic functional somatic syndromes. The implicit objective of the training session is to decrease the counter-attitudes of the physicians towards these patients. The originality of our program is that it mixes the teaching of communication skills and a more introspective teaching of empathy, with a specific focus on the practitioners’ well-being, taking into consideration that they are expected to take care of these patients on a long-term basis. When asked about the impact of the training program on their clinical practice, participants are very positive overall, highlighting three aspects in particular: they feel more competent to give a name to psychosomatic suffering; they recognize the importance of self-compassion for themselves; and they can identify favorable changes in their therapeutic posture.

### Naming psychosomatic suffering

Our finding is in line with recent guidelines on good management of functional somatic syndromes stressing the importance of providing an explanation to the patients about the mechanism of their symptoms [13]. These explanations should “make sense, remove any blame from the patient, and generate ideas about how to manage the symptoms” [33], and also be co-created by the patient and physician [34]. Like other training programs described in the recent literature [17, 18], our program stresses the importance of good communication skills, with a particular focus on the issue of naming chronic psychosomatic suffering. Many studies have shown that finding a name for the symptoms helps the patient feel validated and agree on a treatment plan [35–37]. Consistent with these previous studies, participants expressed that the training program has decreased their uncertainty facing these syndromes and helped them to feel more confident in asserting a functional diagnosis, in giving reassuring explanations about the symptoms. Thus, the physicians felt less pressure to search for a physical cause to the symptoms and could avoid unnecessary investigations. Instead, they learned how to help the patient to live better with their disease, which is consistent with the WONCA recommendations [38].

The crucial issue of naming chronic psychosomatic conditions is an overarching category [39] that points to a shift in the care and understanding of chronic psychosomatic disorders. Understanding the physiopathology and psychopathology can lead to a positive description of the disorder in question, rather than presenting it as a diagnosis of exclusion, as has been the case with psychosomatic disorders in the past. With this knowledge, GPs will be better able to identify clinical syndromes in this area and will be better able to share their understanding with their patients, ultimately helping to reassure them [40, 41]. With a better understanding of the clinical syndrome, GPs and patients are in a better position to adopt a “solution-seeking” attitude, either towards specific treatment options if they exist, as is the case with functional neurological disorders [42], or towards care that allows patients to live better with their health problem. It is important to mention that some participants expressed the view that the name we choose is not in fact very important, but merely a starting point for an open discussion with the patient about the mechanism of the symptoms.

### The importance of self-compassion for physicians

While mindfulness has been extensively studied as a tool for improving doctor-patient relationship [43], compassion meditation is an emerging topic of interest for researchers and teachers in the field of empathy and compassion fatigue [44]. Compassion meditation (or loving-kindness meditation) is a mental training technique, which involves cultivating a benevolent intention towards others, whishing that others be relieved of their suffering. This technique is grounded in mindfulness meditation and also includes a practice of self-compassion, i.e. directing a benevolent intention towards oneself and realizing that our shameful imperfections and sense of failure are also shared by all other human beings (this concept being called common humanity or shared humanity) [45].

Our teaching program contains a short introduction to compassion meditation, including self-compassion. Despite some controversy about the exact delineation of this concept [46], self-compassion is increasingly recognized as a tool for helping caregivers facing difficult medical situations, improving their communication skills and empathy, and preventing compassion fatigue [45, 47]. In our study, we realized that a one-day initiation is not enough for physicians to develop a regular practice of compassion meditation, which requires a great deal of self-discipline and also the support of a practice group, with a sense of belonging to a community that shares common values. Moreover, this type of practice does not fit everybody. However, the collective experience of a common humanity shared in a peer group can already have a profound transformative power on physicians, helping them to free themselves of the guilt they may feel about their failure to cure patients in chronic psychosomatic suffering.

Two years after the training program, participants reported they greatly appreciated the attention given to their well-being as caregivers, which showed them a way to a more compassionate attitude towards themselves and helped them to develop new strategies to take better care of themselves. The components of *self-compassion* and *shared humanity* therefore seem to be the most operational in the teaching of compassionate meditation for participants. This ties in with the thinking developed by Sinclair and colleagues [46], who propose understanding the term *self-compassion* as “a composite of common facets of self-care, healthy self-attitude, and self-awareness […] which likely have a positive effect on the caregiving outcomes that impact healthcare providers and their patients, including compassion”.

At a time when overload and the risk of burnout are a threat to general practitioners, caring for patients with chronic psychosomatic conditions can add to their burden; the evaluation of this training shows that self-care is a strong support in meeting these challenges and improving their own well-being and enjoyment of work.

### Changes in therapeutic posture

In our study, most participants reported that the training program has helped them adopt a therapeutic posture that reconciled their “selfcare” and their “caregiving”. Scientific research has encountered difficulties in demonstrating the effects of interventions on the participants’ professional practice. For example, quantitative literature examining the effects of Balint groups, a common educational format dedicated to psychosomatic approach and focusing on doctor-patient relationship, is scarce and inconclusive [48]. However, the same literature review found that indications of the value of Balint groups was more evident in some qualitative studies [48]. Participants in our study reported a better ability to define their own limits, which is very similar to the ‘knowledge of one’s own limits’ reported in the Van Roy et al. study (2015). Similarly, participants felt better attuned with the patient, echoing the ‘awareness of one’s own and patients’ feelings’ identified in the Van Roy et al. study (2015). Participants also expressed indications of increased sense of competence in the GP-patient encounter as in in the Van Roy et al. study when they reported ‘increased self-confidence and sense of competence’ and ‘feeling more confident to talk about psychosomatic conditions”. Interestingly, participants ventured in modifying their therapeutic objectives possibly indicating the internalization of the new perspective/conceptual framework presented in the program. Changes in their experience of the encounter were also mentioned, perhaps indicating of a maturation of their engagement in the relationship. Participants reported ‘feeling more comfortable during consultations, with more pleasure’ and that they could experience sharing the same human condition as their patients: ‘doctors are human beings like everyone else’. Finally, they were able to take a different pace in the consultation opening up the possibility of a different dialogue that acts as psychosomatic care.

The changes reported by the participants under this heading can be summarized as changes in confidence, practice, and relationship, which shed light on some components we suggest should be included in a training program for chronic psychosomatic disorders. During the first two days, participants were presented with the theoretical underpinnings of functional disorders by specialized faculty, but were also asked to present a case and engage in interactive discussion. We believe that this training combining theory and experience contributed to an increase in participants’ overall confidence based on a better knowledge of physiopathology and psychopathology coupled with their engagement in communicating about the disorder. The changes in practice reported by participants further support the importance of combining cognitive and experiential components in teaching about psychosomatic disorders. We engaged participants in co-constructing a care plan with the patient and worked through case studies, but we also asked participants to be experientially active in role-playing to become more aware of their emotions while communicating with a patient about their chronic psychosomatic condition. We would like to emphasize the importance of emotional involvement in this communication with patients in psychosomatic distress. Communicating about a very often indecipherable condition challenges both parties, potentially keeping them at bay in the encounter. Through self-awareness and group feedback, participants were invited to focus on the emotions and bodily sensations they experienced during role-playing and the practice of self-compassion. They gained a sense of shared humanity with their patients. We believe that communication skills grounded in this human experience contributed to changes in therapeutic attitudes that allowed family physicians to pace consultations differently and feel more comfortable during consultations. These new attitudes can help bridge the gap in the encounter.

### The power of emotional sharing within a group

An aspect that was not anticipated was the power of the group effect: sharing negative feelings and discovering that other physicians also often feel insufficient seemed to have a beneficial effect on participants. Many of them expressed a desire for additional meetings, which led to the formation of a “supervision and meditation practice group”. About half of the participants in the first and second training sessions regularly attend these meetings. We hypothesize that the emphasis on experiential and introspective learning, and the significant personal commitment required in our program allowed this positive group effect to emerge. This tends to confirm the beneficial effect on physician well-being of regular small group meetings with facilitated discussions, reflections, and shared experiences, as demonstrated by West and collaborators [49]. It is also consistent with the findings of Menezes and collaborators [26], who conclude that a sustainable program is better than a single training activity for teaching empathy and compassion to medical students, regardless of the educational setting used.

### Limitations and perspectives

Although the participants did not know the interviewer (AL), one can imagine that they tended to give more positive responses in order to please the other investigators, who are also trainers in the educational program. Practitioners’ improved mental states might be influenced more by the interaction with trainers than by the content of the training. Disentangling the effect of meeting with trainers from the specific effect of training is not straightforward. Both in the focus group and in individual interviews we asked questions on participants’ experience of the course. Only interactions with peers emerged as themes in the analysis of participants’ reports with no specific mentions of the trainers. No specific question addressed appreciation of trainers, however. They would have helped address this limitation. Quantitative measures (such as a compassion fatigue scale, or a self-efficacy scale for example) before and after the training would be interesting to assess the impact of that type of training program on physicians in a future setting. Measures of impact on patient outcomes would also be valuable, but very substantial to implement. Although we met with participants two years after the training and the results of our study showed lasting effects, the longer-term effects of the training program remain uncertain. It is not unlikely that without further complementary professional training, the program may yield only short-term positive effects. In fact, the follow-up of the study indicate that a significant proportion of the participants received additional training in meditation and self-compassion. This finding may suggest a possible secondary effect of the training, however short-term its primary results may have been. Given that general practitioners are requesting more regular sessions of this type, it would be interesting to evaluate which is the lightest training format that would maintain a positive long-term effect on the doctor-patient relationship.

## Conclusion

Our study suggests that a combination of theory, practical communication skills and introspective learning, including self-compassion, could be a promising training framework to improve the therapeutic attitude of physicians when treating patients with chronic functional somatic syndromes. An important proportion of the participating GPs have expressed a desire for continuing medical education with regular sessions that include time for sharing negative feelings about the difficulties in the doctor-patient relationship.

## Electronic supplementary material

Below is the link to the electronic supplementary material.

Supplementary Material 1

Supplementary Material 2

## Data Availability

Data underlying this article cannot be shared publicly for the privacy of individuals that participated in the study. Data will be shared on reasonable request to the corresponding author.

## Declarations

### Research data access policy

The data underlying this article cannot be shared publicly for the privacy of individuals that participated in the study.
