# Supplementary material for: Improving general practitioners’ approaches to functional somatic syndromes: a pilot training program with a focus on compassion and communication
Source: BMC Med Educ. 2025 Jan 18;25:87. doi: 10.1186/s12909-024-06619-0 (PMC11742205; doi:10.1186/s12909-024-06619-0)
Supplement: Supplementary file 1 — Supplementary Material 1 [file 12909_2024_6619_MOESM1_ESM.docx]

**Supplementary material 1 Individual interview guide**

**Professional background and interest in chronic psychosomatic suffering**

Can you tell me about your career in medicine, and what led you to psychosomatic and psychosocial medicine?

What does “chronic psychosomatic suffering” mean to you?

Can you tell me what got you interested in this field?

Do you treat many people with chronic psychosomatic suffering?

What interests you about it and, conversely, what makes it difficult for you?

**Discovery, interest, and experience of training**

How did you find out about the training and what interested you in it?

What impressed you most about the course?

What were the strengths of the course? What could be improved?

**Current management of chronic psychosomatic suffering**

Has the way you deal with chronic psychosomatic suffering changed since you took this course?

Has your understanding of the term “psychosomatic” changed after this training? If so, in what way?

Have you perceived any personal changes as a result of this training?

Have you noticed any changes in the way you diagnose chronic psychosomatic suffering?

Have you noticed any changes in your relations with patients suffering from chronic psychosomatic pain?

Have you noticed any changes in your clinical practices?

During the training course, you discussed the distinctions between different diagnoses of chronic psychosomatic suffering: chronic pain, fibromyalgia, chronic depression, chronic functional digestive disorders, functional neurological disorders, and hypochondria. Do you use this knowledge in your current practice?

- If so, how?
- If not, why not?

During your training, you were introduced to compassionate meditation. How did you experience this introduction to compassionate meditation? What did you think of it?

Did it make any difference to you or your clinical practice?

Do you still use compassionate mediation?

- If not: why not?
- If yes: how and in what context do you use it? With what effects?

The literature shows that there is an increased risk of burn-out or compassion fatigue when dealing with patients in chronic psychosomatic suffering. How do you guard against this?

Have your practices changed as a result of your training?

**If their practices haven't changed:**

What would you have needed for the training to change your practices?

What would you have needed to ensure that what you learned from the training would continue in your practice?

**Summary**

Overall, do you now feel more comfortable diagnosing and managing patients with chronic psychosomatic suffering?

- If so, why? What has changed?

- If no: what would you need?
