# Supplementary material for: Improving general practitioners’ approaches to functional somatic syndromes: a pilot training program with a focus on compassion and communication
Source: BMC Med Educ. 2025 Jan 18;25:87. doi: 10.1186/s12909-024-06619-0 (PMC11742205; doi:10.1186/s12909-024-06619-0)
Supplement: Supplementary file 2 — Supplementary Material 2 [file 12909_2024_6619_MOESM2_ESM.docx]

**Supplementary material 2 Focus group guide**

**Introduction (10 min)**

**Introduction:** Participants were invited to join this focus group after attending the training course *"Chronic Psychosomatic Suffering in Primary Care: Preventing Compassion Fatigue"* in 2019.

We would like to hear about their experience with the training, whether they found it useful, and what they remember from it one year later. Their feedback is valuable and will help us improve the training for future participants.

Reminder of focus group rules: confidentiality, anonymity, and respect for everyone's opinions.

**Round table:** Please introduce yourself in a few words and share why you're here (what motivated you to participate in this focus group).

**Focus Group Questions (1 to 1.5 hours)**

1. What were your expectations when you signed up for this training? What motivated you to enroll?
   - Were your expectations met?
   - What else would you have expected?
   - What changes would you suggest to the training to better meet your expectations?
2. Looking back on the three weekends of training, how did you experience them?
   - What did you gain from it? What do you remember most?
   - What did you like or dislike about the training?
   - What were its strengths and weaknesses?
3. In the evaluation of the training, we asked you what important concept you had retained. Here is the list of the responses we received:
   - co-construction of the diagnosis (5x)
   - I'm not the only one struggling
   - Positive diagnosis
   - Provide explanation
   - Paying more attention to one’s own feelings
   - Be inventive
   - Taking time
   - Co-creation, feedback loop
   - Pain threshold
   - treatment of functional neurological disorders
   - chronic fatigue syndrome

- To what extent are these concepts still important to you, one year after the training?

- Have these notions led to any changes in:

o the way you diagnose chronic suffering?

o your relationships with your chronically suffering patients?

o your clinical practices?

1. In the training evaluation, we asked you what changes might occur in your clinical practice as a result of the training. Here is a list of the answers we received:

- Keep calm (3x)
- going at the patient's pace
- probably a lot of change
- diagnostic exploration
- become aware of my feelings, and take care of them
- more availability for the patient, because less stress about the diagnosis
- taking care of oneself as a caregiver
- integrate compassionate meditation to support myself

- What about today?

- Have you actually observed these changes?

- If so, what has changed concretely? How did it change?
- If not, what was “missing” for it to change?

- Did your understanding of the term “psychosomatic” change after this training?

- Have you observed any other changes?

- Do you still use compassionate meditation today?

- If so, in what situations? With what goals? How often?
- If not, why not?

1. Overall, do you now feel more comfortable diagnosing and managing patients with chronic psychosomatic suffering?

- If so, why? What has changed?
- If not, what would you need?
